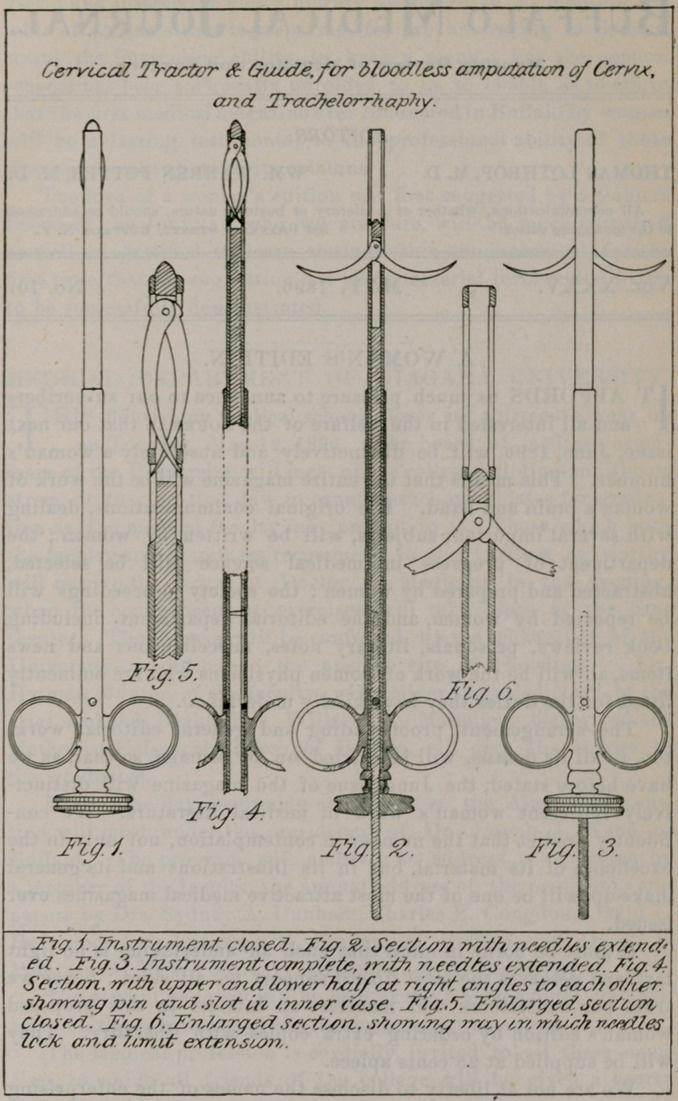# Cervical Tractor and Guide, for Trachelorrhaphy

**Published:** 1896-05

**Authors:** A. H. Macbeth

**Affiliations:** Buffalo, N. Y.; 35 West Eagle Street


### New Instruments.

### CERVICAL TRACTOR AND GUIDE, FOR TRACHELORRHAPHY.

By A. H. MACBETH, M. D., Buffalo, N. Y.

THIS instrument is designed to facilitate amputation of cervix and trachelorrhaphy. I have used it several times and find great advantages over other instruments in use. It is simple and easy to manipulate and makes the operation for which it was intended bloodless and saves much time. Very little need be said about the instrument, as the illustration on opposite page is self-explanatory, showing the instrument in several ways, with the workings and way in which it is made. It is 8½ inches in length and 3-16 of an inch in diameter at the distal end.

I will explain briefly its use: One of the lips of the cervix is drawn down with a bullet forceps, the instrument closed and introduced into the cervical canal a suffieient distance for the needles, when extended, to appear in the anterior and posterior fornices or laterally a short distance above the line to which the operator wishes to cut, stopping the needles as soon as the points appear through the cervical tissues. Then a piece of soft elastic tubing or, better, a rubber elastic ring is placed around cervix above the points of the needles, which serve to retain the rubber ring or tubing in place and cut off the circulation ; the needles extend by means of a screw in the end of the handle, and can be stopped at any stage of the extension, up to two inches, where they lock, but should pass through the cervix barely sufficient to hold the rubber band in place. If extension of the needles be carried too far, injury might result to the vaginal walls and the like. Now the operator has the uterus and cervix under perfect control, with but one instrument, the handles of which serve as a perfect tractor and the stem as a guide to the cervical canal, which is to be preserved.

With the advantages thus obtained, one can easily see that amputation of the cervix, or trachelorrhaphy, would be very much facilitated.

35 West Eagle Street.

## Figures and Tables

**Fig. 1. f1:**